# Anti-Ku myositis presenting with progressive inflammatory myocarditis: diagnostic pitfalls in a fatal case—case report

**DOI:** 10.1093/ehjcr/ytag625

**Published:** 2026-08-31

**Authors:** Ahmed ELBekiey, Mohamed Wahib Zhlawi, Jie Tong, Debashis Roy, Hossam ElGendi

**Affiliations:** Cardiology Department, Princess Alexandra Hospital Trust, Princess Alexandra Hospital, NHS, Hamstel Road, Harlow, Essex CM20 1QX, UK; Cardiology Department, Princess Alexandra Hospital Trust, Princess Alexandra Hospital, NHS, Hamstel Road, Harlow, Essex CM20 1QX, UK; Cardiology Department, Princess Alexandra Hospital Trust, Princess Alexandra Hospital, NHS, Hamstel Road, Harlow, Essex CM20 1QX, UK; Cardiology Department, Princess Alexandra Hospital Trust, Princess Alexandra Hospital, NHS, Hamstel Road, Harlow, Essex CM20 1QX, UK; Cardiology Department, Princess Alexandra Hospital Trust, Princess Alexandra Hospital, NHS, Hamstel Road, Harlow, Essex CM20 1QX, UK

**Keywords:** Case report, Cardiomyopathy, Heart failure, Inflammatory myopathies, Anti-Ku myositis

## Abstract

**Background:**

Anti-Ku antibodies are rare disease of overlap myositis and often signal multisystem involvement. Overt heart failure, however, is seldom reported.

**Case summary:**

A 60-year-old man presented with progressive exertional dyspnoea and chest tightness. Initial echocardiography showed mildly reduced left ventricular ejection fraction (LVEF 49%), and coronary angiography was normal, leading to a diagnosis of MINOCA. One month later, he re-presented with worsening breathlessness, dysphagia, and proximal muscle weakness. Laboratory tests revealed serum creatine kinase 2154-2680 IU/L, troponin 1437 ng/L, and pro-BNP 3290 ng/L. Repeat echocardiography demonstrated dilated ventricles and LVEF 35%. During admission, he developed high-grade AV dissociation requiring dual-chamber pacing and abrupt thrombocytopenia. Cardiac MRI identified a diffuse infiltrative pattern with LVEF 18%. Myositis-specific immunoblot detected high-titre anti-Ku antibodies, and deltoid biopsy confirmed inflammatory myopathy. Despite high-dose corticosteroids, mycophenolate, cyclophosphamide, rituximab and intravenous immunoglobulin, cardiac function deteriorated to LVEF 12%. Recurrent aspiration pneumonia necessitated gastrostomy. Eight months after initial presentation, the patient died following transition to palliative care.

**Discussion:**

This case highlights the danger of assuming that an elevated troponin level is equivalent to myocardial infarction. Non-dynamic troponin elevation with global LV dysfunction and non-obstructive coronaries indicates myocardial injury and warrants investigation for inflammatory and other non-ischaemic causes. Systemic ‘red flags’ such as dysphagia must trigger early multidisciplinary evaluation to avoid fatal diagnostic delay.

Learning points

- Elevated troponin does not equal myocardial infarction. Non-dynamic elevation with global LV dysfunction and non-obstructed coronaries signals myocardial injury, warranting inflammatory workup.
- Extra-cardiac symptoms in unexplained cardiomyopathy should be flagged and require proper investigations for inflammatory and autoimmune workup.
- Anti-Ku myositis could present with severe cardiac-dominant disease first. Unlike other myopathies, cardiac involvement is usually subclinical.

## Introduction

Idiopathic inflammatory myopathies (IIM) are heterogeneous autoimmune diseases primarily affecting skeletal muscles. Anti-Ku antibodies were first described in 1981 as a serological marker of polymyositis–scleroderma overlap syndromes.^1^ They are infrequently detected—in ∼1 in 3500 antinuclear antibody-positive samples—making anti-Ku myositis an uncommon subtype.^2^ Patients typically present with overlapping features of connective tissue disease, such as Raynaud’s phenomenon, arthralgias, sclerodermatous skin changes, proximal muscle weakness, and interstitial lung disease.^2,3^ Idiopathic inflammatory myopathies often involves multiple organ systems; however, clinically overt cardiac disease remains relatively rare, with most cardiac involvement detectable only through subtle subclinical changes.^4^ In contrast, anti-Ku-positive myositis demonstrates a greater propensity for cardiac involvement.^5^ Fulminant cardiomyopathy in anti-Ku myositis is exceptionally rare.^6^

This case presents a patient with infiltrative cardiomyopathy and underscores the diagnostic challenges posed by a condition that affects multiple organ systems. By sharing this uncommon presentation, we aim to enhance recognition of cardiac involvement in anti-Ku myositis and to guide clinicians encountering similar challenges.

## Summary figure

**Figure:**
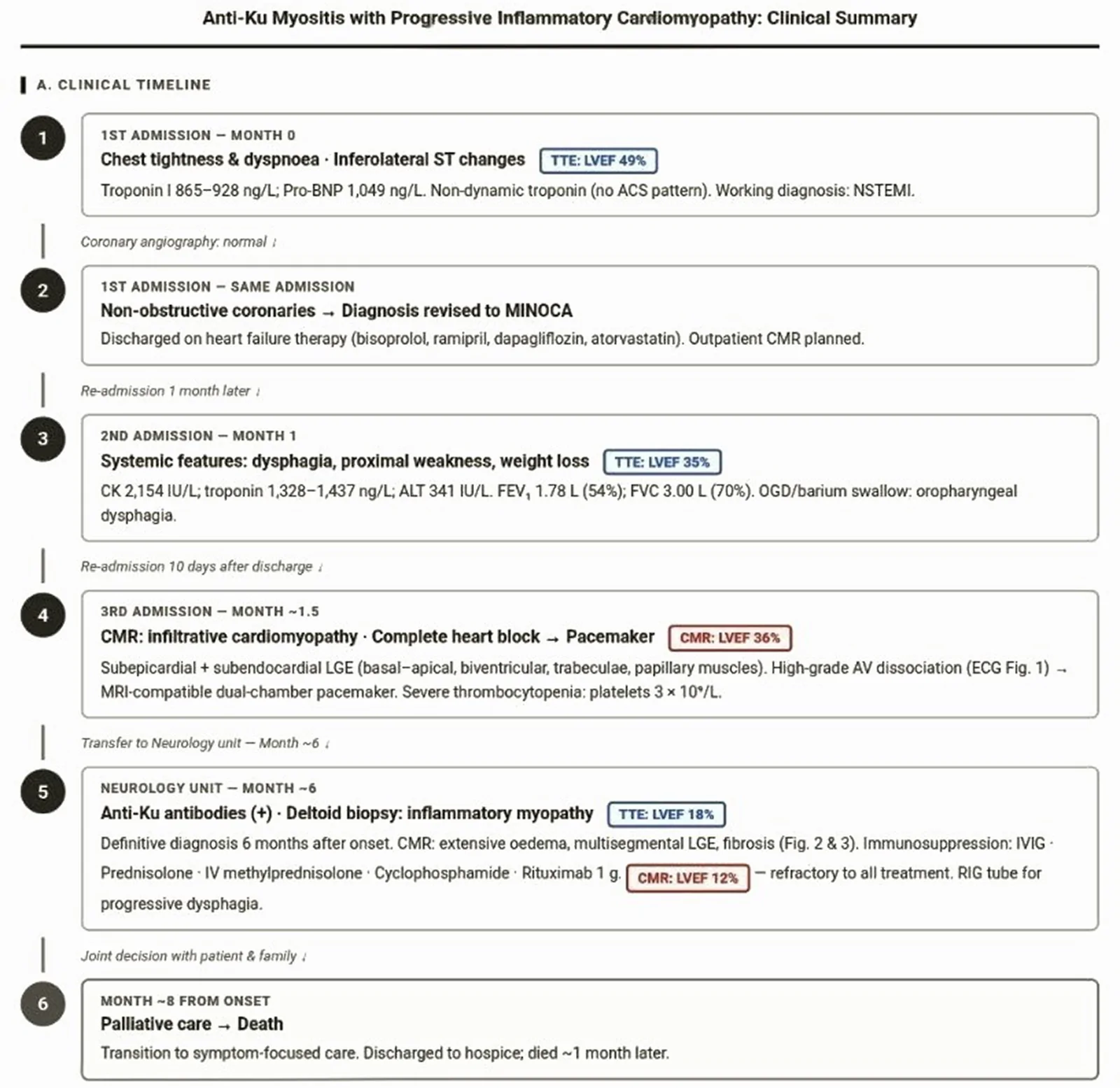


## Case presentation

A 60-year-old male initially presented to the emergency department with a 3-month history of exertional breathlessness and chest tightness. He had no previous significant medical, occupational, or familial history. His 12-lead ECG demonstrated ST-segment depression in the inferolateral leads.

On cardiovascular examination, heart sounds were present without audible murmurs or a gallop rhythm. Chest auscultation confirmed equal bilateral air entry with no added sounds. No significant peripheral oedema was identified. Peripheral pulses were regular and of normal volume bilaterally. Neurological and musculoskeletal assessment, including evaluation of proximal limb muscle power, was unremarkable at this stage.

Initial laboratory investigations revealed elevated cardiac biomarkers: troponin I 865–928 ng/L (reference range < 35 ng/L) and pro-brain natriuretic peptide (pro-BNP) 1049 ng/L (reference range < 300 ng/L) (*Table 1*). A transthoracic echocardiogram (TTE) demonstrated a non-dilated left ventricular (LV) cavity with mildly impaired LV systolic function (LVEF 49%), without regional wall motion abnormalities or significant valvular disease. The working diagnosis was non-ST-elevation myocardial infarction (NSTEMI); however, subsequent coronary angiography revealed non-obstructed coronary arteries. Crucially, there was no significant dynamic rise or fall in troponin levels during the first 48 h—a pattern more consistent with chronic myocardial injury or ongoing inflammation than acute infarction. The patient was discharged with a plan for outpatient cardiac magnetic resonance imaging (CMR) and heart failure pharmacotherapy comprising atorvastatin, bisoprolol, dapagliflozin, and ramipril, all at standard therapeutic doses.

**Table 1:** Timeline of main events and investigations throughout the patient hospital stays

Day of admission	Event
1st admission	Initial presentation with exertional shortness of breath and chest tightness. Elevated troponin levels (865–928 ng/L). Transthoracic echocardiogram (TTE) showing mild LV systolic impairment (LVEF 49.1%). Coronary angiogram showing non-obstructed coronary arteries; diagnosis of MINOCA.
2nd admission: Day 1 (1 month later)	Hospital readmission with progressive breathlessness, dysphagia, fatigue, and weight loss. Persistently elevated troponin (1328–1437 ng/L). Pro-BNP 3290 ng/L. Alanine transaminase 341 IU/L.
2nd admission: Day 5	Follow-up TTE showing dilated LV with worsening systolic function (LVEF 35.1%).
2nd admission: Days 15–20	Oesophagogastroduodenoscopy (OGD) showing hiatus hernia and gastritis. Barium swallow showing no significant oesophageal narrowing. Diagnosed with oropharyngeal dysphagia.
2nd admission: Day 21	Discharge after improvement in dysphagia.
3rd admission: Day 1 (10 days later)	Hospital readmission with persistent productive cough of white phlegm and worsening breathlessness. Elevated troponin levels (1000.0–1270.0 ng/L).
3rd admission: Day 15	Cardiac MRI showing sub-epicardial enhancement in the basal to mid septum, basal inferior wall, and RV insertion points. Sub-endocardial enhancement extended from the mid to apical cavity, with additional involvement of trabeculae and papillary muscles in both ventricles. LVEF 36%.
3rd admission: Day 19	Sigmoidoscopy showing benign rectal polyps.
3rd admission: Day 32	Development of complete heart block requiring dual-chamber pacemaker insertion.
3rd admission: Day 38	Creatinine kinase was tested: 2154 IU/L.
3rd admission: Day 45	PET scan showing FDG-avid consolidation right lower lobe and posterior right upper lobe lung. The appearances were compatible with active infection.
3rd admission: Day 50	Development of severe thrombocytopenia (platelet count 3 × 10^9^/L).
3rd admission: Day 52	Bedside spirometry showing: FEV1 0.82 L (27% predicted) and FVC 1.59 L (41% predicted). This is compared to spirometry done as an outpatient between the first two admissions: FEV1 1.78 L (53.6% predicted) and FVC 3.00 L (70.3% predicted).
3rd admission: Day 78	Transfer to the neurology unit.
Neurology unit: Day 1	TTE showing severely impaired LV function (LVEF 18%).
Neurology unit: Day 3	Anti-Ku antibody test positive.
Neurology unit: Days 5–35	Received IVIG and started oral prednisolone 40 mg daily, later switched to IV methylprednisolone. Treatment included cyclophosphamide and a dose of rituximab 1 g. High-dose IV methylprednisolone pulses were given, then tapered to 30 mg daily.
Neurology unit: Day 37	Cardiac showing severely impaired biventricular function (LVEF 12%).
Neurology unit: Day 39	Liver ultrasound showing mild heterogeneous echotexture.
Neurology unit: Day 55	Decision for palliative care (symptom control only).
Neurology unit: Day 59	Discharge to hospice care. The patient passed away about 1 month later.

One month later, the patient re-presented with progressive breathlessness, dysphagia, fatigue, and weight loss. Clinical examination revealed symmetrical proximal muscle weakness predominantly affecting the shoulder and pelvic girdle musculature, with relatively preserved distal limb power. Oropharyngeal dysphagia to both solids and liquids was confirmed on clinical assessment. Cardiovascular examination demonstrated features consistent with progressive cardiac decompensation, including a more elevated jugular venous pressure and bilateral peripheral oedema compared with the initial presentation. Laboratory investigations are detailed in *Table 1*, showing persistently elevated troponin, elevated pro-BNP, and elevated liver enzymes.

Repeat TTE demonstrated a dilated LV cavity with worsening systolic function (LVEF 35%). Pulmonary function tests revealed significantly compromised ventilatory function with FEV_1_ 1.78 L (53.6% predicted) and FVC 3.00 L (70.3% predicted) (reference range for both ≥80% predicted). Dysphagia was further investigated with oesophagogastroduodenoscopy (OGD) and barium swallow studies, confirming a hiatus hernia with gastritis and no significant oesophageal narrowing, consistent with a diagnosis of oropharyngeal dysphagia. The patient improved with supportive care and was discharged against medical advice on Day 21.

Ten days after discharge, the patient was re-presented with persistent productive cough and worsening breathlessness. Cardiac MRI confirmed dilated left ventricle with moderately impaired systolic function, LVEF 36% (Supplementary material online, *Videos S1* and *S2*). Sub-epicardial enhancement was noted at the basal to mid cavity septum and basal inferior wall. Additionally, sub-endocardial enhancement extended from the mid to apical cavity and further enhancement of the trabeculae and papillary muscles in both the LV and RV. Overall appearances were consistent with an infiltrative cardiomyopathy with main differential diagnosis included myocarditis.

During this third admission, the patient developed high-grade atrioventricular dissociation presenting as symptomatic bradycardia and hypotension, confirmed on ECG (*Figure 1*). An MRI-compatible dual-chamber permanent pacemaker was inserted. The case was reviewed at the inherited cardiomyopathy multidisciplinary team meeting, where further evaluation with PET-CT was recommended alongside formal rheumatology and neurology consultation. A muscle biopsy was advised as the preferred first-line tissue investigation, with myocardial biopsy reserved if skeletal muscle biopsy proved inconclusive.

**Figure 1:**
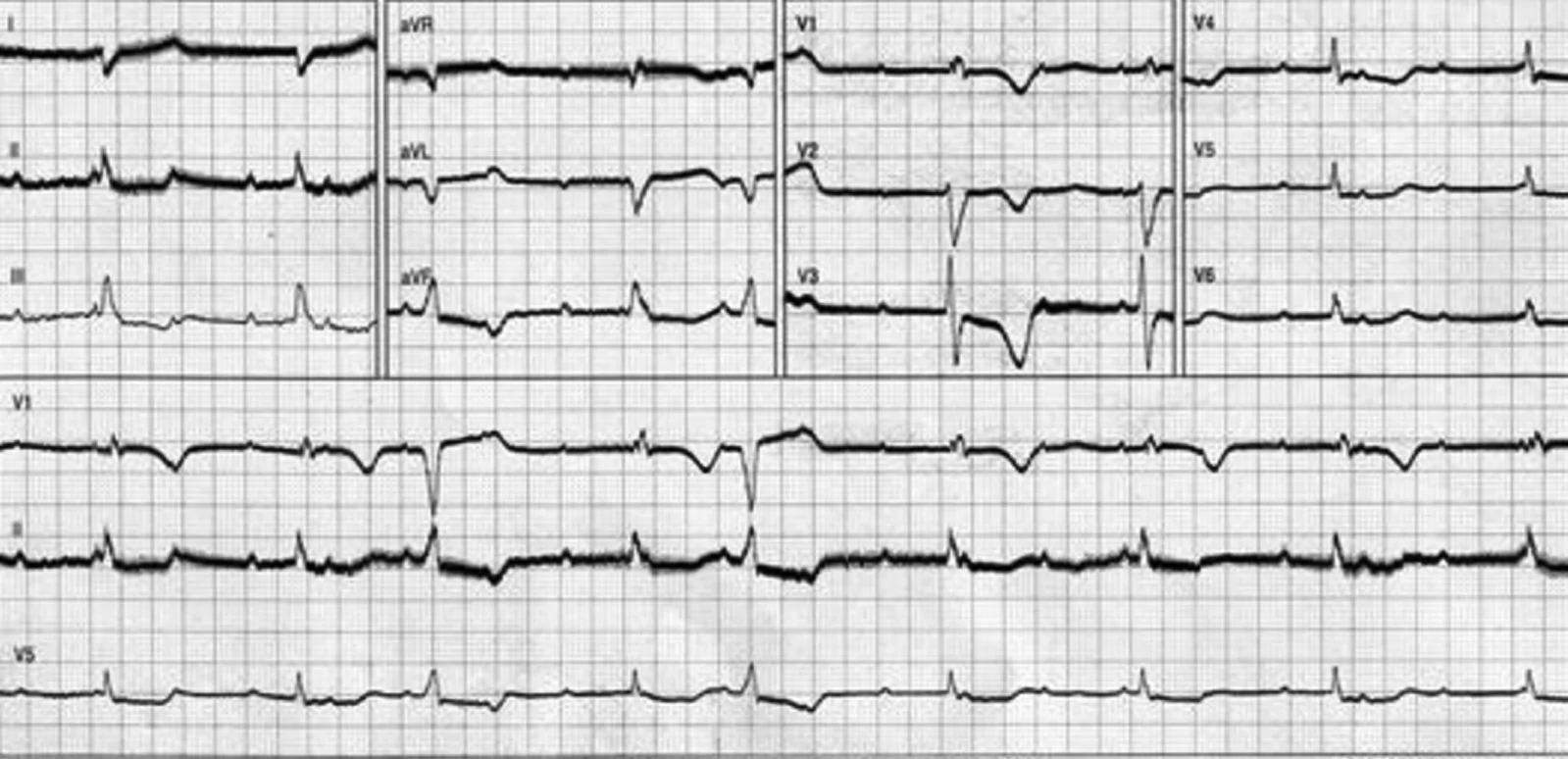
Twelve-lead electrocardiogram at presentation of, showing atrioventricular dissociation with independent atrial and ventricular activity.

The clinical course was further complicated by the development of severe thrombocytopenia, with the platelet count falling to a nadir of 3 × 10^9^/L (reference range 150–400 × 10^9^/L), which significantly limited additional therapeutic options including plasma exchange. Concurrent bedside spirometry demonstrated further respiratory deterioration, with FEV_1_ 0.82 L (27% predicted) and FVC 1.59 L (41% predicted).

On Day 78 of the third admission, the patient was transferred to a specialist neurology unit for further assessment. Repeat TTE at this stage confirmed severe LV systolic dysfunction (LVEF 18%). Comprehensive myositis-specific antibody testing detected high-titre anti-Ku antibodies, and deltoid muscle biopsy confirmed inflammatory myopathy (*Figures 2* and *3*). The definitive diagnosis of anti-Ku myositis was established ∼6 months after initial presentation.

**Figure 2:**
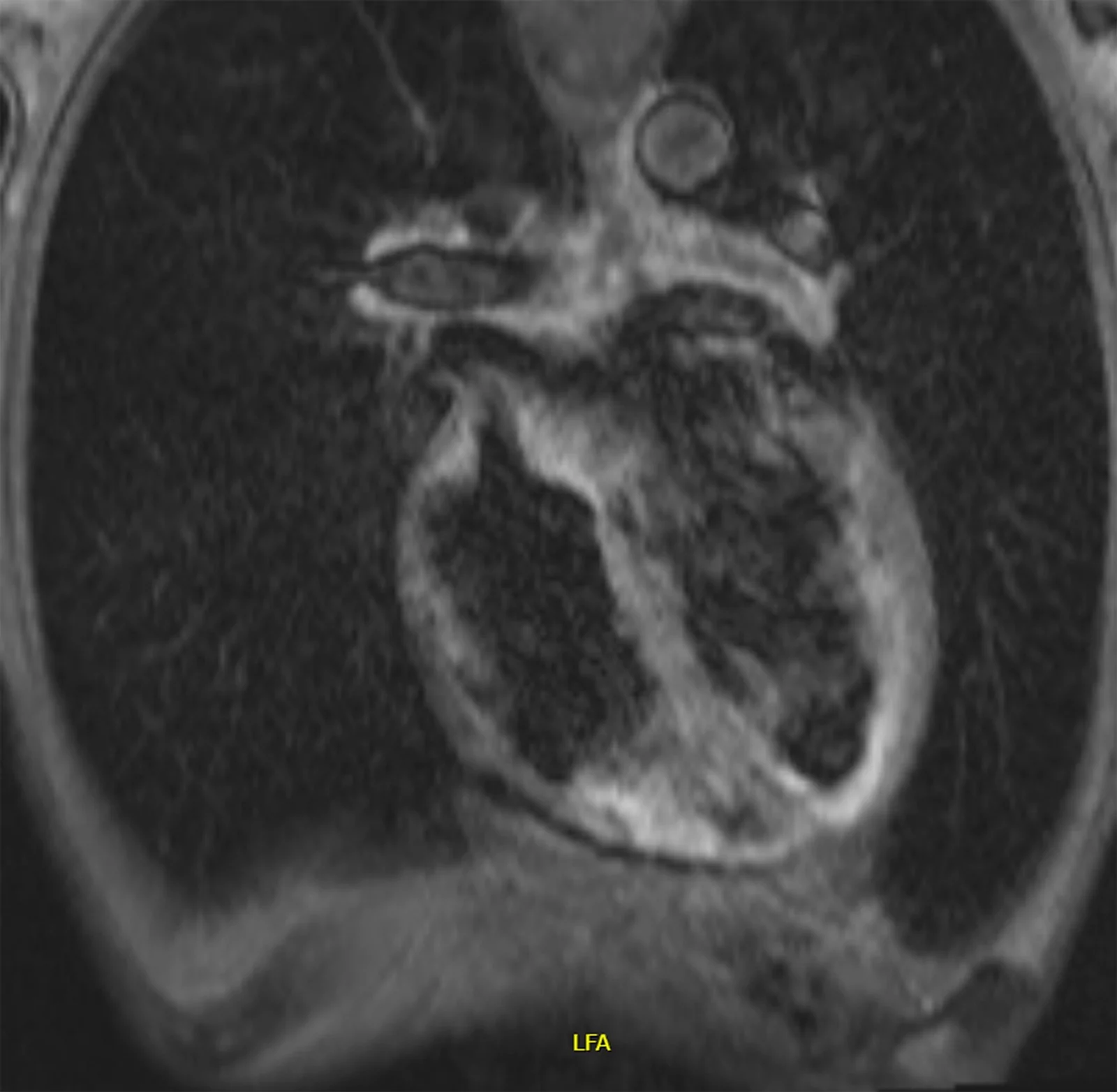
Cardiac MRI, four-chamber late gadolinium enhancement view, showing a dilated left ventricle with extensive sub-endocardial enhancement.

**Figure 3:**
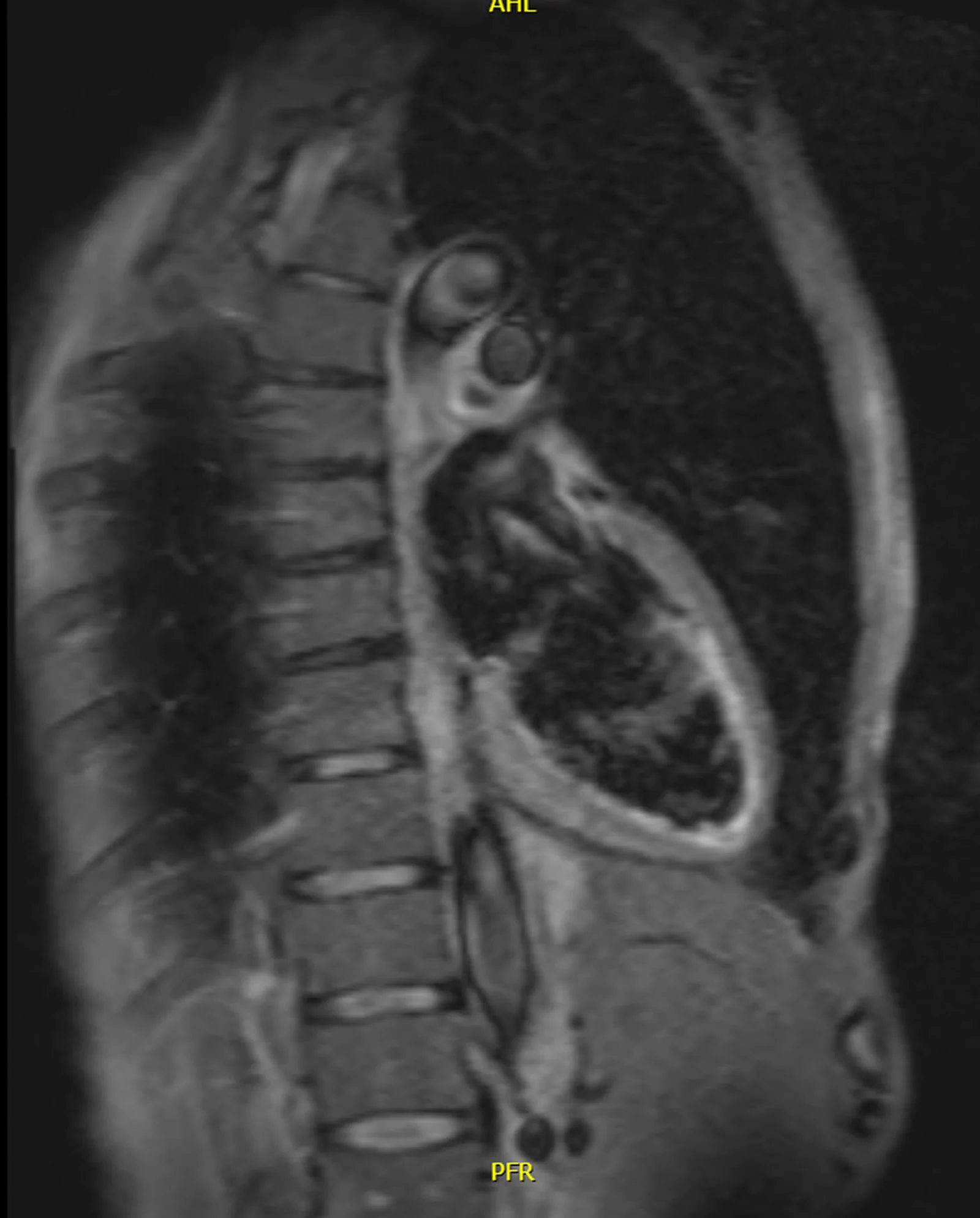
Cardiac MRI, two-chamber late gadolinium enhancement view, showing diffuse, patchy myocardial enhancement.

## Treatment and outcome

Multimodal immunosuppression was initiated upon transfer to the neurology unit and continued over a period of ∼30 days (Days 5–35). Treatment comprised intravenous immunoglobulin (IVIG) administered at a weight-based standard dose over 5 days, followed by oral prednisolone 40 mg daily. In view of progressive deterioration, treatment was escalated to high-dose intravenous methylprednisolone pulse therapy, subsequently tapered to oral prednisolone 30 mg daily. Intravenous cyclophosphamide was administered at institutional protocol-guided doses. Additionally, a single intravenous infusion of rituximab 1 g was given.

Despite this aggressive multimodal immunosuppressive regimen, the patient’s condition continued to deteriorate. Repeat CMR demonstrated severely impaired biventricular function with LVEF declining to 12%. Cardiac magnetic resonance imaging tissue characterization revealed extensive myocardial oedema, LGE in multiple cardiac segments, and features of myocardial fibrosis, consistent with advanced inflammatory cardiomyopathy.

Skeletal muscle strength continued to decline, affecting both proximal and distal muscle groups and progressively impairing swallowing ability, necessitating insertion of a radiologically inserted gastrostomy tube. Respiratory function remained severely compromised and was complicated by recurrent aspiration pneumonia despite gastrostomy feeding.

Following detailed discussions with the patient and his family regarding prognosis and the limited response to aggressive immunosuppression, a joint decision was made to transition to palliative care with a focus on symptom control and comfort. The patient was discharged to a hospice and died ∼1 month later, ∼8 months from his initial emergency department presentation.

## Discussion

### Diagnostic challenges in anti-Ku myositis

Anti-Ku myositis presents unique diagnostic challenges due to its protean clinical manifestations and propensity for cardiac-dominant presentations. High clinical suspicion is required, as cardiac involvement may overshadow skeletal muscle symptoms, which can be subtle or delayed at onset. Overlapping features with systemic sclerosis and lupus further complicate diagnosis, and equivocal initial autoimmune workups may mislead clinicians towards alternative diagnoses.

Dysphagia represents a critical yet under-recognized red flag. A systematic review and meta-analysis reported dysphagia in 36% of patients with IIM, while a Mayo Clinic series identified dysphagia as the presenting symptom in 21% of patients with inflammatory myopathy-associated swallowing dysfunction.^7,8^ Anti-Ku-associated myositis appears to carry an even higher burden of swallowing involvement, with dysphagia reported in 58.8% of cases with available data in one recent cohort.^9^ The constellation of cardiac dysfunction, dysphagia, proximal muscle weakness, and systemic symptoms should prompt early consideration of an underlying inflammatory myopathy.

### Cardiac involvement and diagnostic accuracy

The initial troponin elevation in this case likely re-presented acute myocarditis rather than NSTEMI or MINOCA—a distinction with important management implications. While clinically overt cardiac manifestations occur in fewer than 10% of idiopathic polymyositis cases, anti-Ku-positive patients demonstrate substantially higher risk, with ∼23% developing cardiac complications ranging from subclinical myocarditis to life-threatening arrhythmias and cardiomyopathy. Notably, Rigolet *et al*. observed only pericarditis or conduction blocks—but no dilated or systolic cardiomyopathy—among 30 anti-Ku-positive patients.^10^ Fulminant cardiomyopathy remains exceptionally rare, documented in isolated case reports including a 36-year-old patient who developed an LVEF of 10%–15%, comparable to the present case.^6^

### Management and prognostic implications

Optimal therapy for severe anti-Ku myositis remains incompletely defined. Standard approaches utilize corticosteroids as first-line agents combined with steroid-sparing immunosuppressants such as methotrexate, azathioprine, or mycophenolate mofetil. Severe or refractory disease may warrant cyclophosphamide, rituximab, or IVIG. Plasma exchange may benefit selected cases; however, in this instance, profound thrombocytopenia (nadir 3 × 10^9^/L) precluded this therapeutic option.

Prognosis varies by organ involvement and treatment response. While predominantly muscular disease often responds favourably to immunosuppression, extra-muscular manifestations—particularly cardiac—carry a substantially poorer prognosis, frequently attributable to diagnostic delays. This case illustrates this principle starkly: a 6-month interval from initial presentation to definitive diagnosis allowed progression from mild LV dysfunction (LVEF 49%) to end-stage cardiomyopathy (LVEF 12%), which proved refractory to all therapeutic interventions.

## Conclusion

This case illustrates that an elevated troponin level does not automatically equate to myocardial infarction. Non-dynamic troponin elevation in the setting of global LV dysfunction and non-obstructive coronary arteries should prompt investigation for inflammatory and other non-ischaemic causes of myocardial injury. Comprehensive autoimmune panel testing—including a myositis-specific antibody panel—should be considered in patients with unexplained cardiac impairment, particularly when accompanied by systemic features such as proximal muscle weakness, dysphagia, or respiratory compromise. Improved multidisciplinary awareness among cardiologists, internists, rheumatologists, and neurologists is crucial to minimize delays in referral to specialist teams and to enable early intervention during the potentially reversible phase of disease.

### Recommendations

Elevated troponin does not equal myocardial infarction. Non-dynamic troponin elevation with global LV dysfunction and non-obstructive coronary arteries indicates myocardial injury and should prompt investigation for inflammatory and other non-ischaemic causes.

Symptoms suggesting extra-cardiac involvement (e.g. dysphagia, proximal muscle weakness) in the setting of unexplained cardiomyopathy are a red flag for systemic disease and should trigger immediate serum creatine kinase measurement and comprehensive autoimmune workup.

Anti-Ku myositis can present with fulminant cardiac-dominant disease as the index manifestation, unlike many other inflammatory myopathies where cardiac involvement is typically subclinical.

## Lead author biography


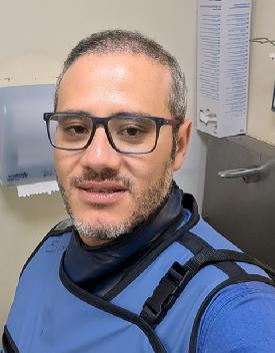


I am working as speciality doctor of cardiology, previously finished cardiology training in Egypt, with experience in cardiology imaging, heart failure, and intervention.

## Supplementary Material

ytag625_Supplementary_Data

## Data Availability

Data will be made available on reasonable request; we have limited access to MRI scans.

## Notes

**Consent:** Written informed consent was obtained from the patient during his lifetime for publication of this case report, including any accompanying clinical images, anonymized data, and videos. The patient was informed that personal clinical details and images would be submitted for publication and that all reasonably identifiable information would be anonymized.
